# Anæsthesia

**Published:** 1871-12

**Authors:** G. Q. Colton

**Affiliations:** New York


### ANÆSTHESIA.

Messrs. Editors.—On returning from Europe a short time since, I picked up your valuable journal, of April, 1870, and noticed a communication under the caption of "Anæsthetics in Surgery—Who Discovered It?" This article, though in the main correct, contains some misstatements, and omits some facts which I think are important to a full understanding of the subject. I agree with you, and with all impartial judges who have examined the subject, in giving the honor of the discovery of anæsthesia to the late Dr. Horace Wells, of Hartford. The statement as to what took place at my exhibition, given in Hartford on the 10th of December, 1844, is correct, though as to what followed is not quite so accurate. The writer says: "'I have a tooth,' said Dr. Wells, 'little decayed, sufficient to be filled, and *it shall be pulled!* We will prepare the gas. I will take it, and *you shall pull the tooth to-morrow!*' said he to Dr. Riggs, who assented to this proposition, and said, 'It is fair to begin on ourselves;' and on that the surgeons separated for the night, with fixed determination and high hopes.

"In the morning, December 11, 1844, the gas was prepared. At half past ten o'clock A. M. Dr. Wells took the dentist's chair in his own office, at the corner of Main and Asylum streets—the office now occupied by Dr. Taft. * * But Dr. Wells took the pipe of the gas-bag in his mouth, and it was kept there till his hands fell lifeless, and his head drooped. Then in an instant Dr. Riggs fastened his forceps to the left upper molar and drew it out. Dr. Wells did not move a muscle. Holding the tooth up to the spectators, Dr. Riggs said, 'You see!' In a moment Dr. Wells opened his eyes, blew the blood from his mouth, and asked, 'Is that my tooth?' 'It is,' said Dr. Riggs."

The author of the above must have drawn upon his imagination for some of these details. He conveys the impression—without saying it in so many words—that my agency in this matter ended with the exhibition in the hall, and that either Dr. Wells or Dr. Riggs made and administered the gas for this first operation under an anæsthetic. The facts are as follows: At the close of the exhibition Dr. Wells came to me and stated that Colonel Cooley felt no pain while the effects of the gas lasted, and asked why a tooth could not be extracted without pain, &c. I replied that I did not know, as the thought had never occurred to me. I then related the fact that at my exhibition in Bridgeport, a short time previous, a young man broke his finger in striking another while under the influence of the gas, but said he felt no pain till the effects of the gas had passed off. Dr. Wells *then and there* decided to try the experiment on himself, in having a tooth extracted, and asked me if I would administer the gas to him for the purpose. I did so the next day, forenoon. Dr. Wells was delighted with the success of the experiment, *and asked me to instruct him how to make the gas, which I did.*

I claim no special honor in all this, but what little is due me should not be given to another. Dr. Wells immediately introduced the use of the gas into his dental practice, with entire success, and continued it for a year, or till December, 1845, when he went to Europe for the benefit of his health. Dr. Wells died on the 24th of January, 1848, and before the dental or surgical profession had recognized the value and efficiency of the gas as an anæsthetic. I think that Dr. Riggs never made the gas at this time, and only used it in connection with Wells—else why should he abandon it for fifteen years, or till I revived it in May, 1863?

After the death of Wells, the general verdict was that the gas had been tried by him and proved a failure. Not being a dentist or surgeon myself, I had no use for the gas, though I often referred to the experiments of Wells, and claimed that the gas *was* an anæsthetic, and Wells was the author of the discovery of anæsthesia. But these claims were never generally admitted till after I had revived the gas and demonstrated its value. It lay dead for fifteen years. The credit of this revival and practical demonstration I claim, and I feel confident that the profession will award it to me.

The proposal which you advocate, to erect a monument to the memory of Dr. Wells, meets my hearty approval, and I shall be glad to unite with all those who have been benefited by his great discovery in contributing to that object.

Yours truly, G. Q. Colton.

New York, November 13, 1871.

